# Patient‐level and organizational‐level factors influencing in‐hospital falls

**DOI:** 10.1111/jan.15254

**Published:** 2022-04-20

**Authors:** Jinhyun Kim, Eunhee Lee, Yoomi Jung, Hyunjeong Kwon, Sunmi Lee

**Affiliations:** ^1^ College of Nursing Seoul National University Seoul South Korea; ^2^ School of Nursing/Research Institute of Nursing Science Hallym University Chuncheon Gangwon‐do South Korea; ^3^ Korea Armed Forces Nursing Academy Daejeon South Korea

**Keywords:** clinical competence, nurse staffing, nurses, patient falls, patient safety

## Abstract

**Aim:**

In‐hospital fall is one key safety issue in a healthcare setting. Although healthcare providers apply several strategies for preventing falls, falls still occur in hospitals. The aim of this study was to investigate patient‐level and organizational‐level factors influencing in‐hospital falls.

**Design:**

A multicentre retrospective observational study.

**Methods:**

This study used the national healthcare database and supplemented with organizational data obtained through a survey. Data extraction and survey were conducted between July and August 2020. A mixed‐effect logistic regression model was used to analyse factors influencing in in‐hospital falls.

**Results:**

A total of 43,286 patients admitted in 86 hospitals were included in this study. Fall rate was 0.85 per 1000 days. Length of stay was significantly longer for fall patients than for no‐fall patients. Patient‐level factors (including age, mobility impairment and surgery) and organizational‐level factors (including nurse staffing and proportion of new nurses) were significant factors influencing in‐hospital falls.

**Conclusion:**

Since in‐hospital falls increase economic burden to patients, we should consider various fall prevention strategies to reduce falls. For a strategy to be applied stably to patients, organizational factors must be supported.

**Impact:**

Proactive fall management in acute settings is essential to ensure patient safety. Considering that the number of patients with fall risk is increasing due to ageing, organizational factors should be supported to provide quality nursing care for fall risk patients. Therefore, nurse leaders should primarily ensure an appropriate level of nurse staffing. They also need to make efforts to strengthen clinical competency of nurses.

## 1 INTRODUCTION

Accidental fall during hospitalization is one of key safety issues that should be prevented. Although falls and fall‐related injuries occur infrequently (Everhart et al., 2014; Krauss et al., 2007), they can cause health problems and increase the economic burden of patients (Baris et al., 2018; Zecevic et al., 2012). Thus, healthcare providers need to assess the risk of falls and use preventive strategies during hospitalization. Although various fall prevention devices have been developed recently for use in clinical settings, falls still occur in hospitals. They have not declined sharply yet. It is known that individual factors and organizational factors could influence in‐hospital falls. However, few multilevel studies including several organizational factors such as nurse staffing and nurse career or using fall prevention devices have been conducted. Therefore, the objective of this study was to investigate both individual and organizational factors influencing patient falls.

## 2 BACKGROUND

### 2.1 Risk factor for in‐hospital falls

#### 2.1.1 Patient factors

Patients' risk factors for in‐hospital fall have been identified in several studies (Cox et al., 2015; Hartikainen et al., 2007; Kim et al., 2019). They could be categorized into advanced age, medical conditions, medications and impaired mobility. Older adults aged 65 years and over had higher risk of falls, not only for inpatients, but also for community residents (Moreland et al., 2020; Sherrington et al., 2019). It has been reported that 27.5% and 10.2% of the older people have at least one fall in a year and a fall‐related injury respectively (Moreland et al., 2020). Accordingly, most instruments for assessing the risk of falls included patient's age. In addition, clinical practice guidelines for the prevention and management of falls in the older people are being applied to clinical practice. Several studies have shown that prevention strategies are effective (Montero‐Odasso et al., 2021). Not only age, but also patient's health status can influence the risk of falls. In particular, since patients' mobility is highly correlated with in‐hospital falls, diseases such as Parkinson's disease that can directly affect patient mobility can increase patient's fall risk (Pelicioni et al., 2019; Silva de Lima et al., 2017). Moreover, diseases and medications known to cause changes in blood pressure can also significantly increase the risk of falls (Hart et al., 2020; Sibley et al., 2014). As individual risk factors might not be modifiable immediately, several strategies should be applied in clinical practice to prevent in‐hospital falls of patients with risk factors. Organizational factors such as nurse staffing and hospital environment should also be considered. Hence, an investigation of the risk of falls requires evaluation of not only individual factors, but also organizational factors.

#### 2.1.2 Hospital factors

Among hospital factors, nurse staffing plays a significant role in preventing or managing falls in clinical settings. The longer the patients were administered nursing care by registered nurses (RNs), the lower the patients' risk of falls (Kim et al., 2019; Kim & Bae, 2018; Staggs & Dunton, 2014). Hence, adequate nurse staffing is essential to improve patient outcomes and to reduce the risk of falls.

Although absolute nurse staffing is closely related to patient safety, the competency of nurse is also a factor related to patient safety such as fall (Ayton et al., 2017; Fehlberg et al., 2020). Nurses' competency such as decision‐making and situation awareness is associated with nurse's experience (Nibbelink & Brewer, 2018). Newly graduated nurses who have a short experience in clinical practice might not provide high‐quality care.

Recently, the hospital environment is being improved to decrease falls in hospitals. Various fall preventing devices such as motion sensors, monitors and wearable devices have been developed to prevent falls of the older people (Bowles et al., 2015; Rantz et al., 2013; Sun & Sosnoff, 2018). While these devices have been applied to community‐dwelling older people for assessing their fall risk with effectiveness in preventing the falls (Kristoffersson et al., 2021; Silva de Lima et al., 2017; Sun & Sosnoff, 2018), these are underutilized in acute care setting because of concerns about their reliability, cost and effectiveness. As patients with high risk of falls require close observation, these assistive devices may need to be used to aid in patient monitoring, taking into account time constraints of healthcare providers. However, the effectiveness of the device on fall prevention should be primarily evaluated for application in patients admitted to acute care settings.

### 2.2 Nurse staffing in Korea and integrated nursing care

Although adequate nurse staffing is an essential factor among several safety indicators affecting in‐hospital falls (Kim & Bae, 2018; Kouatly et al., 2018), hospitals in South Korea cannot secure adequate nurses for inpatient care due to nursing shortage (Cho et al., 2016). A nurse should take care of more than 15 patients in South Korea (Cho et al., 2016). This number is much higher than those in the United States and several European countries (Aiken et al., 2012). To increase nurse staffing, Korea has continued to increase nursing school enrolment to meet the projected demand for registered nurses. In addition, a new integrated inpatient care system, also known as integrated care system, was implemented to improve patient outcomes in 2013 by doubling nursing staff in existing nursing units (Kim et al., 2019). Regardless of the medical department, patients can be admitted to the integrated care unit with an additional fee. Nurse staffing levels in integrated care units have been various according to types and characteristics of hospitals (Kim et al., 2017). Tertiary hospitals have the highest nurse staffing levels, followed by general hospitals and semi‐hospitals. One RN takes care of 5–7 patients in a tertiary hospital, 7–12 patients in a general hospital and 10–16 patients in a semi‐hospital (Kim et al., 2017). A nurse assistant (NA takes care of 30–40 patients in a tertiary hospital and 25–40 patients in a general hospital or semi‐hospital (Kim et al., 2017). Increasing nurse staffing in the integrated care system can lead to better satisfaction and improve patient safety (Kim et al., 2019).

To meet the increased demand for nurses in an integrated nursing unit, many newly graduated nurses are assigned to such unit. Since some studies have reported that newly graduated nurses have low levels of competency for patient safety (Ayton et al., 2017; Labrague & De Los Santos, 2020), nursing units with high proportion of newly graduated nurses could not provide high‐quality care. For better patient outcome, not only adequate nurse staffing, but also organizational factors such as nurse experience and skill mix should be considered.

## 3 METHODS

### 3.1 Aim

This study aimed to investigate both individual and organizational factors influencing in‐hospital falls.

### 3.2 Design

We conducted a multicentre retrospective observational study to examine factors influencing in‐hospital falls. Factors influencing patient falls during hospitalization were investigated primarily using the database from the National Health Insurance (NHI). In addition, we conducted a survey of organizational variables not included in the NHI database.

We surveyed 86 hospitals accounting for 17.1% of total hospitals. These hospitals had integrated care units. Since we conducted a mailed survey in August 2020 due to COVID‐19 outbreak, the response rate was not higher than expected.

### 3.3 Participants

This study included 43,286 patients who were admitted to integrated care units of 86 hospitals from January 2020 to March 2020. We included only patients who were hospitalized and discharged during this period. We excluded those who were hospitalized for less than 3 days or more than 30 days.

### 3.4 Data collection

We primarily used the database from the NHI and conducted a survey of organizational variables not included in the NHI database. Data extraction from the NHI database and survey were all conducted between July and August of 2020.

The survey was conducted with 522 hospitals having integrated care units in South Korea for investigating careers of nurses and the number of fall prevention devices. We explained the purpose and the content of survey to the nursing administrative department. A structured questionnaire was distributed to all hospitals with integrated care units by mail. Questionnaires were collected from 86 of 522 hospitals.

For building the analytic dataset, we first combined organizational datasets in the NHI database and the survey. Next, we extracted patients' data in the NHI database only for hospitals corresponding to the first step. Patients' data included basic data collected at admission and health data collected daily during hospitalization.

We collected organizational data which included hospital type, ownership, location, number of RNs and NAs from the NHI database, and number of fall prevention devices and the proportion of newly graduated RNs from a survey. According to hospital type, not only the structure such as bed size, medical cost and staffing, but also patients' characteristics differed. Areas where hospitals are located are classified into metropolitan and non‐metropolitan.

Nurse staffing in integrated care units was distinguished by hospital type. The nurse‐to‐patient ratio for RN staffing ranged from 1:5 to 1:7 for a tertiary hospital, 1:7 to 1:12 for a general hospital, and 1:10 to 1:16 for a semi‐hospital. NA staffing ratio ranged from 1:25 to 1:40 for all hospital types. Hospitals could select a suitable nurse‐to‐patient ratio in the range set by hospital type in consideration of the number of patients and characteristics of patients. They could change the nurse‐to‐patient ratio as needed. The nurse‐to‐patient ratio is the same for all integrated units in the same hospital. In addition, hospitals must allocate appropriate nurses according to the selected nurse‐to‐patient ratio. The number of patients in charge per nurse should not exceed that ratio. We transformed the nurse‐to‐patient ratio into hours per patient day (HPPD) calculated by dividing 24 h in a day by the number of patients in charge of a single nurse. Most hospitals used a pressure and motion sensing pad among fall prevention devices. This study analysed whether pressure and motion sensing pads were installed on more than 10% of the total number of beds. In this study, the sensing pad is a device to prevent falls that may occur when moving in bed. Thus, it should be particularly applied to patients with limited bed mobility among patients at risk of falls. According to a previous study, 50% patients were older people and 20% of patients had partial or complete limitation in mobility (Kim et al., 2019). Thus, we categorized hospitals into two groups: (1) hospitals with pressure and motion sensing pads installed for more than 10% of the total number of beds; and (2) hospitals with pressure and motion sensing pads installed for less than 10% of the total number of beds. The proportion of newly graduated RNs was calculated as the percentage of RNs with less than 1 year of total career to the total number of RNs.

We extracted patients' data from NHI database. Age, gender and primary disease for hospitalization at admission were collected. The primary disease was identified using the ICD (International Statistical Classification of Diseases)‐10 codes. Health data were collected daily from admission to discharge, which included respiratory care, drainage maintenance, intravenous (IV) therapy, and activities of daily living (ADL) dependency. Respiratory care was classified into three categories: no treatment, oxygen apply and ventilator care. Drainage maintenance was defined as whether the patient had a drain tube or not. IV therapy, which was related to fall occurrence, included four medications: inotropics, antiarrhythmics, anticoagulants and opioids. ADL dependency was assessed in four areas: bed mobility, transfer, eating and toilet use. It was determined to be independent (score 0), partial dependency (score 1) or full dependency (score 2). Total score of ADL dependency ranged from 0 to 8. We included all fall cases that occurred during hospitalization regardless of injury. Fall rate was presented as falls per 1000 patient days.

### 3.5 Ethical consideration

This study received ethics approval for both the use of NHI database and a survey from the institutional review board (IRB) of the author's university. As NHI database could be extracted as de‐identified data, the requirement of informed consent of patients was waived by the IRB. We only extracted variables previously requested with the consent of the NHI. The survey was conducted after obtaining consent from the nursing department.

### 3.6 Data analysis

Characteristics of organizations and patients were summarized using descriptive statistics. Nurse staffing was reported by nursing assistant‐to‐patient ratios and HPPD by RNs or NAs. Box plots were used to present the total duration of stay according to falls and hospital type. Student's *t*‐test was used to assess the difference in lengths of stay according to fall occurrence.

We extracted hospital data first, followed by data extraction for patients who were admitted to these hospitals. As final analytic data consisted of two levels, which violated independent assumption of a standard logistic regression, we used a mixed‐effects logistic regression model to identify factors affecting patient falls.

Mixed‐effects logistic regression including both fixed and random effects are useful for modelling intra‐cluster correlation in structures such as two‐level, longitudinal and panel data (Hedeker, 2003). In this study, observations in the same hospital were correlated because they shared a common hospital‐level random effect. In‐hospital falls were generally associated with the hospital environment. However, this study did not consider hospitals themselves to be a fixed effect. Thus, the mixed‐effects model was suitable for this study, which was analysed with a multi‐level sample.

The two‐level model in this study included a series of independent clusters depending on a set of random effects *u*_*j*_, for *j* = 1…*n* (hospitals) with hospital *j* consisting of *i* = 1… *n*_*j*_ (patients). Since the dependent variable was fall occurrence with a binary value reported by each patient, the logistic cumulative distribution function (H) was used in this study (Equation 1).

(1) $$\mathrm{Pr}\left(y_{\mathrm{ij}}=1|x_{\mathrm{ij}},u_{j}\right)=H\left(x_{\mathrm{ij}}\beta +z_{\mathrm{ij}}u_{j}\right),$$

where *x*_*ij*_ is covariates of fixed effects, including patient factors and hospital factors, and *z*_*ij*_ is covariates of random effects not estimated directly in model parameters but summarized according to variance components. Stata SE version 14.2 was used for all statistical analyses.

### 3.7 Validity and reliability

In this study, the National Health Insurance (NHI) database, a representative and reliable population‐based database in Korea, was used. In particular, data of integrated care unit from NHI database were collected by healthcare workers regularly trained using the instrument developed by Song et al. (2010). The instrument by Song et al. (2010) has been verified for its validity and reliability in various studies. In particular, the study by Cho et al. (2020) confirmed the validity of this instrument for measuring nursing needs in patients admitted to an integrative care unit.

## 4 RESULTS

### 4.1 Patients' characteristics

This study included 43,286 patients who were admitted to integrated nursing care units from January 2020 to March 2020. Their characteristics are shown in Table 1. According to hospital type, 27,525 patients were hospitalized to general hospitals, 8273 patients were admitted to tertiary hospitals and 7448 patients in semi‐hospitals. The proportion of females was higher than that of males in all hospital types. The average age was 60.47 years. The proportion of older people patients aged 65 years and older was the highest in general hospitals, followed by those in tertiary hospitals and semi‐hospitals. The primary disease of patients at admission also differed according to the type of hospital. While patients in tertiary hospitals were most often hospitalized for neoplasm, those in general and semi‐hospitals were hospitalized mostly for musculoskeletal diseases. Approximately 50% of patients were admitted for a surgical procedure. According to hospital types, the admission rate in semi‐hospitals was more than 70%, which was the highest. Most patients did not receive respiratory care. However, fewer than 10% were administered either oxygen or received ventilator support. The proportion of patients treated with drainage was 5.3%–13.6%, which was not high. As intravenous (IV) treatment could affect falls, the number of patients treated with opioids was the highest, followed by those treated with inotropics, antiarrhythmics and anticoagulants. In particular, patients admitted to semi‐hospitals also showed a high rate of IV opioid use due to a high rate of surgical procedure. ADL dependency showed a linear pattern according to hospital type. The proportion of partial dependency was higher than that of full dependency in all areas. In each area, needing assistance for transfer had the highest proportion of patients (20.9%), followed by toilet use (15.4%), bed mobility (10.7%) and eating (9.5%).

**TABLE 1:** Patients' characteristics in an integrated care unit located in Korea

		Total	Tertiary hospitals^a^	General hospitals^b^	Semi‐hospitals^c^	F/x^2^	*p*‐value (scheffe)
		(*n* = 43,286)	(*n* = 8273)	(*n* = 27,525)	(*n* = 7488)		
Gender	Male	20,378 (47.1)	4125 (49.9)	12,881 (46.8)	3372 (45.0)	39.17	<0.001
	Female	22,908 (52.9)	4148 (50.1)	14,644 (53.2)	4116 (55.0)		
Age	Mean (SD)	60.47 (18.42)	61.05 (17.64)	61.51 (18.58)	56.01 (18.01)	270.82	<0.001
	<65 years	23,936 (55.3)	4443 (53.7)	14,516 (52.7)	4977 (66.5)		(a, b > c)
	≥65 years	19,350 (44.7)	3830 (46.3)	13,009 (47.3)	2511 (33.5)		
Diseases	Musculoskeletal disease	13,593 (31.4)	933 (11.3)	7678 (27.9)	4982 (66.5)	8919.34	<0.001
	Neoplasm	4692 (10.8)	2453 (29.7)	2186 (7.9)	53 (0.7)		
	Circulatory disease	2783 (6.4)	533 (6.4)	2196 (8.0)	54 (0.7)		
	Respiratory disease	3597 (8.3)	621 (7.5)	2631 (9.6)	345 (4.6)		
	Others	18,621 (43.0)	3733 (45.1)	12,834 (46.6)	2054 (27.4)		
Operation	Yes	19,725 (45.6)	4042 (48.9)	10,272 (37.3)	5411 (72.3)	2942.48	<0.001
	No	23,561 (54.4)	4231 (51.1)	17,253 (62.7)	2077 (27.7)		
Respiratory care	No	40,766 (94.2)	7540 (91.1)	25,915 (94.2)	7311 (97.6)	354.67	<0.001
	Oxygen therapy	2388 (5.5)	663 (8.0)	1548 (5.6)	177 (2.4)		
	Ventilator care	132 (0.3)	70 (0.8)	62 (0.2)	0 (0.0)		
Drainage maintenance		3040 (7.0)	1124 (13.6)	1452 (5.3)	464 (6.2)	682.38	<0.001
Medication	Inotropics	577 (1.3)	170 (2.1)	344 (1.2)	63 (0.8)	47.99	<0.001
	Antiarrhythmics	196 (0.5)	58 (0.7)	133 (0.5)	5 (0.1)	36.63	<0.001
	Anticoagulants	430 (1.0)	201 (2.4)	222 (0.8)	7 (0.1)	244.93	<0.001
	Opioids	14,074 (32.5)	2396 (29.0)	7059 (25.6)	4619 (61.7)	3543.31	<0.001
ADL score	Mean (SD)	0.73 (1.54)	1.15 (1.94)	0.68 (1.48)	0.45 (1.05)	458.28	<0.001 (a > b > c)
Bed mobility	Partial	3078 (7.1)	943 (11.4)	1769 (6.4)	366 (4.9)	783.86	<0.001
	Full	1573 (3.6)	581 (7.0)	925 (3.4)	67 (0.9)		
Transfer	Partial	6557 (15.1)	1542 (18.6)	3823 (13.9)	1192 (15.9)	989.59	<0.001

Full	2495 (5.8)	1001 (12.1)	1310 (4.8)	184 (2.5)		
Eating	Partial	3314 (7.7)	857 (10.4)	2074 (7.5)	383 (5.1)	339.61	<0.001

Full	770 (1.8)	81 (1.0)	664 (2.4)	25 (0.3)		
Toilet use	Partial	4304 (9.9)	1185 (14.3)	2450 (8.9)	669 (8.9)	849.90	<0.001

Full	2392 (5.5)	842 (10.2)	1435 (5.2)	115 (1.5)		

Sign of inequality (e.g. a,b>c) is the result of post hoc test. So the significance of post hoc test is <.05.

### 4.2 Hospital characteristics

Among 522 hospitals operating integrated care units in South Korea, 86 were included in this study. More than 50% of hospitals were general hospitals. Most (76.7%) hospitals were privately owned. Hospitals located in the non‐metropolitan were included slightly more than those in the metropolitan area. Nurse staffing in integrated nursing care system differed according to hospital type. As nurse staffing in tertiary hospitals was the highest in all types of hospitals, the average HPPD was the highest in tertiary hospitals. Standard staffing procedures constituted the highest proportion in all types of hospitals. Standard staffing ratios were 1:5, 1:10 and 1:12 for tertiary hospitals, general hospitals and semi‐hospitals respectively. Unlike RN staffing, NA staffing was similar for all types of hospitals, which had only three staffing levels: 1:25, 1:30 and 1:40. Thus, HPPD by NA did not differ substantially among hospital types. Among nurses who worked at integrated nursing units, newly graduated nurses who worked in a year accounted for 18%–22%. The proportion of newly graduated students was the highest in tertiary hospitals, followed by those in general hospitals and semi‐hospitals. The use of pressure and motion sensors, the device used to prevent patients' falls, was the highest in tertiary hospitals but the lowest in general hospitals (Table 2).

**TABLE 2:** Hospital characteristics

		Total	Tertiary hospitals	General Hospitals	Semi‐hospitals
		(*n* = 86)	(*n* = 9)	(*n* = 49)	(*n* = 28)
Ownership	Public	20 (23.3)	3 (33.3)	13 (26.5)	4 (14.3)
	Private	66 (76.7)	6 (66.7)	36 (73.5)	24 (85.7)
Area	Metropolitan	36 (41.9)	3 (33.3)	18 (36.7)	15 (53.6)
	Non‐metropolitan	50 (58.1)	6 (66.7)	31 (63.3)	13 (46.4)
RN staffing	1:5	2 (2.3)	2 (22.2)		
	1:6	6 (7.0)	6 (66.7)		
	1:7	2 (2.3)	1 (11.1)	1 (2.0)	
	1:8	19 (22.1)		19 (38.8)	
	1:10	34 (39.5)		27 (55.1)	7 (25.0)
	1:12	22 (25.6)		2 (4.1)	20 (71.4)
	1:14	1 (1.2)			1 (3.6)
	HPPD, mean(SD)	2.61 (0.65)	4.11 (0.43)	2.64 (0.34)	2.09 (1.19)
NA staffing	1:25	18 (20.9)	0 (0.0)	13 (26.5)	5 (17.9)
	1:30	58 (67.4)	6 (66.7)	32 (65.3)	20 (71.4)
	1:40	10 (11.6)	3 (33.3)	4 (8.2)	3 (10.7)
	HPPD, mean(SD)	0.81 (0.10)	0.73 (0.10)	0.83 (0.10)	0.81 (0.10)
% of new graduated nurses (SD)		0.20 (0.14)	0.22 (0.09)	0.21 (0.14)	0.18 (0.15)
Pressure and motion sensors	10% and more	21 (24.4)	4 (44.4)	10 (20.4)	7 (25.0)
	Less than 10%	65 (75.6)	5 (55.6)	39 (79.6)	21 (75.0)

### 4.3 Fall rate and average length of stay in case of fall

The average fall rate was 0.85 cases per 1000 patient days. Based on hospital type, the fall rate was 0.48 cases per 1000 patient days in tertiary hospitals, the lowest among all types but the highest in general hospitals. Moreover, fall occurred at 6.71 days after hospitalization. It occurred during hospitalization for 6–7 days in all hospital types. Approximately 50% of patients had hospital falls in 5 days of hospitalization (Table 3).

**TABLE 3:** Fall rate and length of stay and place in case of fall

	Total	Tertiary hospitals	General hospitals	Semi‐hospitals
	(*n* = 237)	(*n* = 9)	(*n* = 49)	(*n* = 28)
Fall rate per 1000 patient days				
Mean (Sd)	0.85 (0.90)	0.48 (0.50)	1.04 (0.98)	0.63 (0.77)
Min–Max	0.00–4.41	0.00–1.64	0.00–4.41	0.00–3.92
Length of stay in case of fall				
Mean (SD)	6.71 (5.84)	7.82 (6.12)	6.56 (5.86)	6.56 (5.59)
Min–Max	1–28	2–28	1–26	1–22
% of occurrence in 5 days	55.3	42.8	57.8	52.8

### 4.4 Length of stay by fall and its influencing factors

**FIGURE 1:**
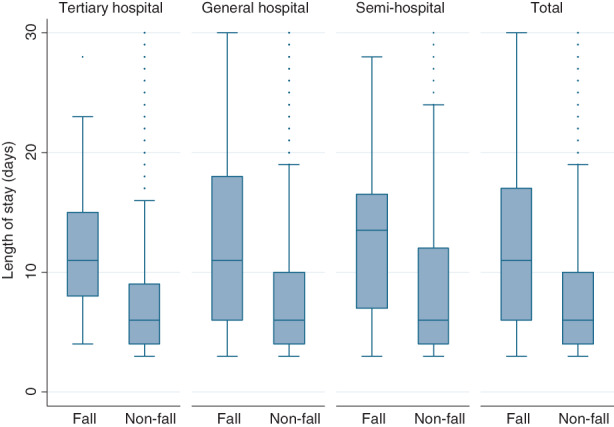
Distribution of fall rate varies with hospital type and length of stay. *Note*: Average length of stay was 8.05 days in non‐fall patients, and 12.56 days in fall patients, which varied significantly by fall occurrence (*t* = 12.041, *p* < 0.001). Due to fall occurrence, the length of stay was extended by 4.43 days in tertiary hospitals (*t* = 4.511, *p* < 0.001), 4.47 days in general hospitals (*t* = 9.976, *p* < 0.001), and 4.59 days in semi‐hospitals (*t* = 4.658, *p* < 0.001)

Patients who experienced fall events during hospitalization had significantly longer hospital stays than those who did not (Figure 1). The average length of stay among non‐fall patients was 8.05 days. It was 12.56 days for fall patients, which was significantly different based on fall occurrence (*t* = 12.041, *p* < 0.001). Due to falls, the length of stay was extended by 4.43 days in tertiary hospitals (*t* = 4.511, *p* < 0.001), 4.47 days in general hospitals (*t* = 9.976, *p* < 0.001) and 4.59 days in semi‐hospitals (*t* = 4.658, *p* < 001).

Mixed‐effect logistic regression analysis was conducted with two models. Model 1 included only individual factors. Model 2 included individual and organizational factors (Table 4). Multicollinearity between independent variables was evaluated using variance inflation factor (VIF), which was less than 3 in both model 1 and mode 2. In model 1, age, transfer dependency and operation were factors significantly influencing falls in this study. The older the patient, the higher the odds of a fall (OR = 2.44, *p* < 0.001). Among the four areas of ADL dependency, only transfer dependency, which was highly relevant to falls, was included. Patients who were partially dependent on transfer were more likely to experience falls during hospitalization (OR = 2.26, *p* < 0.001). In case of patients fully dependent on transfer, the risk of falls was not significantly higher than those without dependency. The risk of falls was lower in patients who had surgery than in those without surgery (OR = 0.58, *p* = 0.005). Various IV medications were not significant factors associated with patients' falls in this study.

**TABLE 4:** Mixed‐effects logistic regression of falls based on individual and organizational factors

Variables	Model 1				Model 2			
	OR	*p*‐value	95% CI		OR	*p*‐value	95% CI	
Individual factors								
Gender (base: men)	0.99	.953	0.76	1.29	0.98	0.881	0.75	1.28
Older people	2.44	<0.001	1.81	3.29	2.42	<0.001	1.79	3.27
Disease (base: musculoskeletal disease)								
Neoplasm	1.07	0.794	0.64	1.79	1.32	0.302	0.78	2.24
Circulatory disease	0.57	0.093	0.30	1.10	0.61	0.130	0.32	1.16
Respiratory disease	0.95	0.861	0.57	1.60	0.97	0.916	0.58	1.63
Others	0.83	0.295	0.58	1.18	0.88	0.489	0.62	1.26
Surgery	0.58	0.005	0.39	0.85	0.58	0.007	0.40	0.86
Respiratory care (base: no)								
Oxygen therapy	0.65	0.148	0.36	1.16	0.66	0.160	0.37	1.18
Ventilator care	1.62	0.517	0.38	7.01	1.66	0.497	0.38	7.18
Drainage maintenance	1.17	0.555	0.70	1.97	1.21	0.465	0.72	2.04
ADL_transfer (base: free)								
Partial dependency	2.26	<0.001	1.66	3.07	2.33	<0.001	1.71	3.17
Full dependency	1.64	0.062	0.98	2.75	1.76	0.032	1.05	2.96
IV medication								
Inotropics	0.80	0.707	0.25	2.58	0.81	0.719	0.25	2.61
Antiarrhythmics	1.57	0.539	0.37	6.58	1.60	0.520	0.38	6.72
Anticoagulants	1.80	0.274	0.63	5.13	1.95	0.215	0.68	5.57
Opioids	0.83	0.357	0.55	1.24	0.85	0.414	0.57	1.26
Organizational factors								
HPPD by RN					0.41	0.016	0.20	0.85
HPPD by NA					0.13	0.103	0.01	1.50
% of new graduated RNs					7.32	0.007	1.72	31.20
Pressure and Motion Sensors (base: less than 10%)					1.11	0.703	0.66	1.86
Hospital types (base: tertiary hospitals)								
General hospitals					0.63	0.437	0.20	2.01
Semi‐hospitals					0.41	0.264	0.08	1.97
Private ownership					0.90	0.698	0.53	1.53
Located at metropolitan					1.29	0.247	0.84	2.00
Random effect (variance)								
Variance (95% CI)	0.53 (0.30–0.95)				0.35 (0.17–0.68)			
log likelihood	−1374.83				−1366.75			
Wald chi(p)	105.80 (<0.001)				126.86 (<0.001)			

In model 2, individual factors that were statistically significant were similar to results of model 1. Furthermore, patients fully dependent on transfer were at a higher risk of falls than those without a transfer dependency. Among organizational factors, RN‐HPPD and the proportion of newly graduated RNs were strongly correlated with falls. Patients in hospitals with a higher HPPD by RN were significantly less likely to fall (OR = 0.41, *p* = 0.016). The higher the rate of newly graduated RNs, the higher the odds of a fall (OR = 7.32, *p* = 0.007). The pressure and motion sensors were not significant factors affecting falls in this study.

## 5 DISCUSSION

The fall rate in this study was estimated to be 0.85 cases per 1000 patient days, which was lower than rates reported in previous studies (Kim et al., 2019; Staggs & Dunton, 2014; Tzeng et al., 2012). We could not generalize this result to all hospitals in Korea because this study only targeted integrated care units with an increase in nurse staffing. However, as the inpatient system in Korea is changing in the direction of the overall spread of an integrated care unit (Kim et al., 2017), the fall rate is expected to decrease in all hospitals in Korea.

Among patient factors, older people patients showed a higher risk of falls, consistent with most other studies (Esain et al., 2017; Lawlor et al., 2003). Since the integrated care unit in Korea is an acute care setting, the proportion of the older people was 44.7%, which was not higher than that in a long‐term care setting. The rate of in‐hospital falls in this study was also lower than rates reported in other studies (Quigley et al., 2012; Vassallo et al., 2009). As older people patients are vulnerable to falls and fall‐related injuries due to comorbid diseases, cognitive impairment, and impaired mobility (Montero‐Odasso et al., 2021), it is essential to provide prevent care for the older people with fall risk regardless of healthcare settings. In addition to age, patient's ADL was another strong influencing factor affecting in‐hospital falls. Patients with partial dependency on transfer had a significantly higher risk of falls than independent patients in both models 1 and 2. This finding suggests that partially dependent patients are more likely to move without help and are highly likely to fall over assistance devices than fully dependent patients (Lawlor et al., 2003; Pi et al., 2016). Therefore, partially dependent patients admitted to the hospital require assistance of not only RN, but also non‐RN staff during movement.

Among procedures and medications, patients admitted to surgery had a lower risk of falls than those admitted for medical treatment. Surgical patients are less likely to sustain falls because of their low mobility dependency, consistent with a previous study reporting that the fall rate in a medical unit was higher than that in a surgical unit (Staggs & Dunton, 2014). While several medications such as antiarrhythmics, opioids, inotropics, and anticoagulants could increase the risk of falls and fall injuries (Hart et al., 2020; Yoshikawa et al., 2020), we did not find any significance associations between these drugs and fall occurrence. Except opioids, as only a few patients receive these medications, there might be limitations in analysing the relationship between medications and patient fall in this study. In addition, some studies did not show clear a correlation between treatment with these medications and falls (Hartikainen et al., 2007; Lawlor et al., 2003; Sterke et al., 2008). It is known that anticoagulant is associated with fall‐related injuries rather than falls (Hartikainen et al., 2007).

Among hospital factors, RN‐HPPD was a significant factor influencing patient falls in this study, corroborating findings of other studies (Kim et al., 2019; Kouatly et al., 2018; Lake & Cheung, 2006; Staggs & Dunton, 2014). The longer the nursing hours provided by RNs, the lower the risk of falls. Despite a significant increase in nurse staffing in the integrated nursing unit, the HPPD by RNs and NAs of 2.61 and 0.81, respectively, was still extremely low compared with that in other countries such as the Unites States and Australia (Everhart et al., 2014; Lake et al., 2010). Therefore, the level of RN staffing in Korea might need to be increased further to enhance the patient outcome. The non‐RN staffing level was not a significant factor affecting patient falls in this study, consistent with several previous studies (Kim et al., 2019; Lake et al., 2010). However, appropriate staffing in NA is just as important as deploying RN staff to reduce in‐hospital falls as patients with partial or full mobility impairments may be at an increased risk for in‐hospital falls if they are unable to receive mobility assistance.

In this study, not only RN staffing, but also the proportion of newly graduated nurses was a significant influencing factor. New graduated nurses who are transitioning to practice have low competencies involving patient safety as well as falls (Murray et al., 2018) and risk management (Johnstone & Kanitsaki, 2007). Although several studies have reported that a transition program for newly graduated nurses is needed for better outcomes for nurses and patients (Ackerson & Stiles, 2018; Van Camp & Chappy, 2017), the training period for new nurses is very short in Korea. No additional staff might be assigned during that period. Therefore, a high proportion of newly graduated nurses could affect patient safety.

The rate of device utilization such as pressure and motion sensors was not a significant factor for falls in this study. Various fall prevention devices have been developed and used for community residents or patients admitted in long‐term care facilities. However, few studies corroborated their preventive role (Kristoffersson et al., 2021; Silva de Lima et al., 2017; Sun & Sosnoff, 2018). Moreover, there is a lack of studies evaluating its effectiveness in acute care setting. Thus, a further study is needed to investigate effects of various devices, especially for patients who were admitted in an acute care setting.

Factors influencing in‐hospital falls are varied and complex. They include not only the individual level, but also the organizational level. In addition, most patient factors such as age and ADL are not modifiable. Patients with fall risks are likely to continue to increase due to ageing. Thus, organizational factors such as nurse staffing and devices should be supplemented to prevent and manage in‐hospital falls for patients with risk factors.

In‐hospital falls extended the length of stay (LOS) by more than 4 days across all hospital types. Prolonged LOS due to falls regardless of fall‐related injuries have been corroborated by many previous studies (Dunne et al., 2014; Morello et al., 2015). However, the difference in LOS according to fall in the present study was not larger than those in previous studies. Since patients with a fall‐related injury had a greater increase in LOS compared with those who sustained minor falls without injury (Morello et al., 2015), short difference might be attributed to the small number of serious injuries in this study.

### 5.1 Limitations

This study has some limitations due to the use of patients' data from a secondary database. We could not include all variables reported in previous studies in our mode. Although previous fall is one of critical factors influencing in‐hospital falls, we could not include this variable in the model because the database did not include that variable. In case of medications, there was a limit to determine their associations with falls because the proportion of patients was extremely small. Fall‐related injuries can induce a greater impact on patients rather than simple falls. According to the guidelines of NHI, all falls ranging from minor falls to serious falls with injuries were reported. However, the severity was not classified. Therefore, we could not investigate the degree of injuries caused by falls.

## 6 CONCLUSION

Our study showed that patient factors (including age, mobility impairment and surgery) and organizational factors (including RN staffing and proportion of newly graduated nurses) could significantly affect patient falls. To prevent in‐hospital falls, various fall prevention strategies are needed to manage patients at risk. In addition, organizational factors should be considered because patient factors are not modifiable in preventing patient falls. Therefore, nurse leaders should ensure an appropriate level of nurse staffing to provide quality nursing care and achieve better patient outcomes. In addition, as the high proportion of newly graduated nurses could affect patient safety, nurse leaders should provide systematic training program for newly graduated nurses and try to secure additional nurses during the transition period of new nurses.

## CONFLICT OF INTEREST

The authors have no conflict of relevant to this study to disclose.

## AUTHOR CONTRIBUTIONS

Jinhyun KIM and Eunhee LEE designed and directed the project. Eunhee Lee, Hyunjeong Kwon and Sunmi LEE extracted the data from the NHI database. Yoomi JUNG conducted the survey. Eunhee Lee and Jinhyun KIM analysed the data and worked on the manuscript. All authors discussed the results and contributed to the final manuscript.

7

### PEER REVIEW

The peer review history for this article is available at https://publons.com/publon/10.1111/jan.15254.
